# A CROSS-CULTURAL ANALYSIS OF PREFERENCES AND ATTITUDES TOWARD ADVANCED DIRECTIVES IN TAIWAN AND THAILAND

**DOI:** 10.1093/geroni/igae098.3057

**Published:** 2024-12-31

**Authors:** Hui-Ching Weng, Duan-Rung Chen, Krungkraipetch Kiiti, Inchai Puangtong

**Affiliations:** National Cheng Kung University, Tainan City, Tainan, Taiwan (Republic of China); National Taiwan University, Taipei, Taipei, Taiwan (Republic of China); Burapha University, Mueng, Chon Buri, Thailand; Burapha University, Mueng, Chon Buri, Thailand

## Abstract

**Introduction:**

Advance directives (ADs) are pivotal in healthcare planning, indicating preferences for medical treatment in critical conditions. This study examines ADs attitudes and experiences between Taiwan and Thailand, highlighting cultural influences on end-of-life care.

**Method:**

Employing pairing matching, the study selected 259 matched pairs from an initial Taiwanese pool of 1488 and a Thai sample of 268 based on gender, age, education, and medical work background. Demographics included 72.2% females, with an average age of 32.91 (range 18-72), 92.3% holding a bachelor’s degree or higher, and 95% working in health-related fields.

**Results:**

The comparison revealed no significant differences in the perceived necessity of ADs at the current life stage or in the preference for familial decision-making during hospitalization. However, significant disparities emerged in several areas, including comfort in discussing death (Taiwan 95.8%, Thailand 84.6%) and willingness to discuss end-of-life care (Taiwan 96.1%, Thailand 91.5%). A substantial gap was found in the belief regarding peers’ consideration of ADs, with 84.6% of Taiwanese versus 49% of Thais believing their age group would consider such directives. Furthermore, all eight aspects related to the desired qualities of healthcare agents showed significant differences, with Taiwanese respondents valuing trust, communication, understanding, and support more highly than their Thai counterparts.

**Conclusions:**

The study underscores cultural differences in attitudes towards advance directives between Taiwan and Thailand, particularly in discussions about end-of-life care and qualities desired in healthcare proxies. These findings highlight the need for culturally tailored approaches to enhance awareness and acceptance of advance directives in both regions.

